# SARS-CoV-2 Omicron BA.1 Challenge after Ancestral or Delta Infection in Mice

**DOI:** 10.3201/eid2811.220718

**Published:** 2022-11

**Authors:** Mariana Baz, Nikita Deshpande, Charlie Mackenzie-Kludas, Francesca Mordant, Danielle Anderson, Kanta Subbarao

**Affiliations:** World Health Organization Collaborating Centre for Reference and Research on Influenza, Melbourne, Victoria, Australia (M. Baz, N. Deshpande, K. Subbarao);; University of Melbourne Peter Doherty Institute for Infection and Immunity, Melbourne (C. Mackenzie-Kludas, F. Mordant, D. Anderson, K. Subbarao);; Victorian Infectious Diseases Reference Laboratory, Melbourne (D. Anderson)

**Keywords:** COVID-19, coronavirus disease, SARS-CoV-2, severe acute respiratory syndrome coronavirus 2, viruses, respiratory infections, zoonoses, vaccine-preventable diseases, Omicron BA.1, challenge, ancestral, mice, neutralizing antibodies, K18-hACE2 mice, SARS-CoV-2 variants, cross-protection, immunogenicity, Australia

## Abstract

We assessed cross-reactivity to BA.1, BA.2, and BA.5 of neutralizing antibodies elicited by ancestral, Delta, and Omicron BA.1 SARS-CoV-2 infection in mice. Primary infection elicited homologous antibodies with poor cross-reactivity to Omicron strains. This pattern remained after BA.1 challenge, although ancestral- and Delta-infected mice were protected from BA.1 infection.

The SARS-CoV-2 Omicron variant (B.1.1.529, BA.1 sublineage) emerged nearly 2 years after the ancestral strain was identified (*1*). The Omicron BA.1 variant contains ≈50 mutations in the spike protein (*2*), resulting in substantial antigenic change. The strain was more infectious than prior variants of concern (VOCs) and escaped immunity, causing infections in persons who were previously vaccinated with ancestral strain–based vaccines (*3*) or infected with the ancestral virus or Delta (B.1.617.2) VOC. Since January 2022, additional Omicron sublineages (BA.2 to BA.5) have been detected worldwide. BA.4/BA.5 have identical spike proteins, most similar to BA.2, with additional spike mutations (*4*).

We sought to mimic the human scenario and selected a mouse model from available animal models (*5*) to assess the cross-reactivity of neutralizing antibody elicited by ancestral, Delta, and BA.1 viruses and to assess the effect of primary homologous and heterologous infection on secondary infection with the Omicron BA.1 strain. We also compared antibody cross-reactivity to BA.2 and BA.5 in serum samples from mice infected with ancestral, Delta, and BA.1 strains.

We first compared the associated illness, mortality rates, and kinetics of replication of 10^4^ 50% tissue culture infectious dose (TCID_50_) of SARS-CoV-2/Australia/Vic/01/20 (ancestral strain–like), SARS-CoV-2/Australia/Vic/18440/2021 (Delta), and SARS-CoV-2/Australia/NSW/RPAH-1933/2021 (Omicron BA.1) strains in 7- to 9-week-old female K18hACE2 transgenic mice (Appendix Figure). We infected groups of 15 K18hACE2 mice with intranasally delivered ancestral, Delta, or Omicron BA.1 strains by using a low dose of each virus (10^2^ TCID_50_), selected so that the mice would survive primary infection (Figure, panel A). We mock-infected 15 mice with phosphate-buffered saline (PBS). We collected blood on day 27 after primary infection and then challenged mice with 10^4^ TCID_50_ of Omicron BA.1 virus. We collected lungs and nasal turbinates (NTs) 2 and 4 days after challenge; we weighed and monitored 5 mice per group for clinical signs for 14 days (Figure, panel B). We collected blood samples on day 28 after Omicron BA.1 challenge (day 56 from primary infection).

**Figure:**
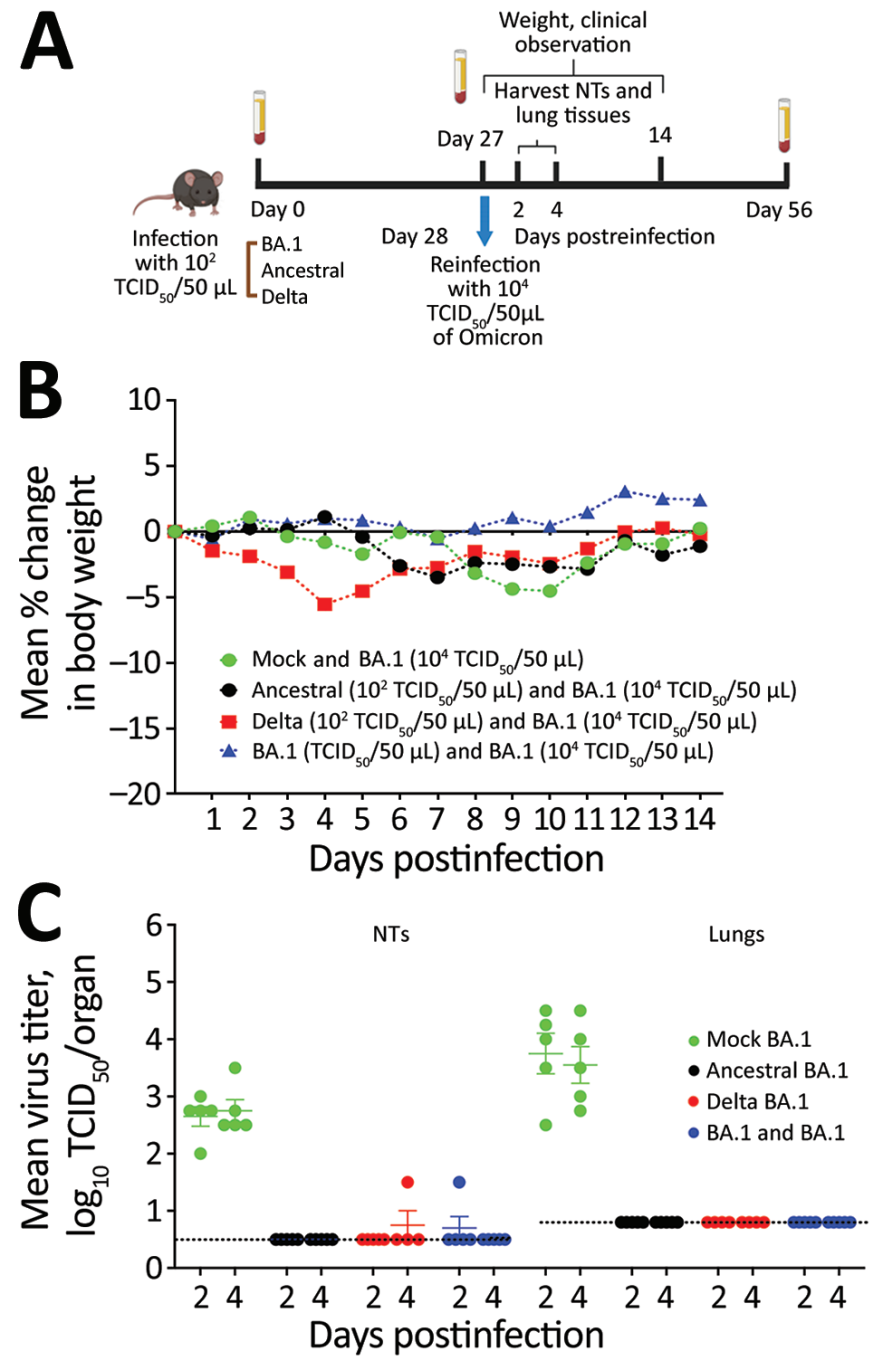
Primary infection with ancestral, Delta, or Omicron BA.1 SARS-CoV-2 strains as protection in mice from BA.1 reinfection. A) Flowchart of 6- to 8-week-old female hACE2K18 transgenic mice who received primary infection with low doses (10^2^ TCID_50_) of Omicron BA.1, ancestral, or Delta viruses and were reinfected with a higher dose (10^4^ TCID_50_) of BA.1. B) Weight loss in mice reinfected intranasally with 50 μL containing 10^4^ TCID_50_ of Omicron on day 28 after primary infection with each SARS-CoV-2 strain. Animals were monitored daily for weight loss, and deaths were recorded over a period of 14 days. Mice were euthanized when they lost 20% of their original bodyweight. C) Replication kinetics of Omicron BA.1 virus in mice after reinfection with 10^4^ TCID_50_/virus. Virus titers in the NTs and lungs of 5 mice per group euthanized on days 2 and 4 postinfection are expressed as log_10_ TCID_50_/mL (NTs) and log_10_ TCID_50_/organ (lungs). Horizontal bars represent mean titers, and symbols represent titers from individual mice. The dashed horizontal line indicates the lower limit of detection, 10^0.5^ TCID_50_ per mL for the NTs and 10^0.8^ TCID_50_ per organ for lungs. NTs, nasal turbinates; TCID_50_, 50% tissue culture infectious dose.

After primary infection, all Omicron BA1–infected mice survived without major weight loss, but 1 ancestral strain–infected and 5 Delta-infected mice died during days 8–13. After challenge with 10^4^ TCID_50_ of Omicron, all mice, including the PBS group (naive control), survived without weight loss. The control group had mean virus titers of 10^2.6^ (day 2) and 10^2.7^ (day 4) in NTs and 10^3.7^ (day 2) and 10^3.5^ (day 4) TCID_50_/organ in lungs after Omicron BA.1 challenge. 

Consistent with other reports (*6*), we found the titers of BA.1 to be lower than those for ancestral and Delta viruses (Appendix Figure, panel C). Virus was not recovered from the tissues of mice challenged with BA.1 that had prior primary infection with ancestral, Delta, or BA.1 viruses (Figure, panel C), except 1 mouse in each of the ancestral and Delta primary infection groups.

The homologous responses were strongest to ancestral (geometric mean titer [GMT] 709), followed by Delta (GMT 129), and were lowest to BA.1 (GMT 83) (Table). The low titer neutralizing antibody response to Omicron BA.1 infection is probably attributable to less robust replication of BA.1 virus in mouse tissues (Appendix Figure, panel C). Mice recovered from primary BA.1 infection were fully protected from rechallenge with the higher dose of BA.1, and no boost in homologous neutralizing antibody titers occurred (day 56 GMT 62).

**Table:** Homologous and heterologous serum neutralizing antibody titers on days 27 and 56 after primary and secondary SARS-CoV-2 infection in hACE2K18 transgenic mice*

Primary infection, 10^2^ TCID_50_	Secondary infection, 10^4^ TCID_50_	Serum neutralizing antibodies (GMT) against indicated virus after primary and secondary infection				
		BA.1	BA.2†	BA.5†	Ancestral	Delta
BA.1	BA.1	**83/62**	10/10	10/10	7‡/7‡	7‡/8‡
Ancestral	BA.1	34‡/27‡	10/10	10/10	**709/1,338**	90‡/>440‡
Delta	BA.1	16/60	10/35	10/53	55‡/124‡	**129/>453**

*Bold indicates homologous titers. GMT, geometric mean titer; TCID_50_, 50% tissue culture infectious dose. †Lower limit of detection in indicated assays is 10. In other assays, the lower limit of detection is 5. ‡Serum samples from different sets of 5 mice from the group were tested on days 27 and 56.

Primary Omicron BA.1 infection did not induce heterologous neutralizing activity against ancestral, Delta, BA.2, or BA.5 viruses (Table). In contrast, primary ancestral infection elicited an 8-fold reduced titer against Delta and 21-fold reduced titer against the BA.1 virus, and primary Delta infection elicited a 2-fold reduced titer against ancestral strain. None of the mice first infected with BA.1, ancestral, or Delta viruses developed neutralizing antibodies against BA.5.

Despite the absence of detectable BA.1 virus in the respiratory tract tissues after secondary infection in mice previously infected with ancestral or Delta (Figure, panel C), we observed a boost in homologous GMTs 1,338 (ancestral) and >453 (Delta), and cross-reactive neutralizing antibody titers GMTs >440 (ancestral) and 124 (Delta), and vice versa (GMTs of 27 and 60, respectively), with no improvement in cross-reactivity to BA.1. Mice first infected with Delta and rechallenged with BA.1 had low but detectable neutralizing antibody titers against BA.5 (Table).

Our observations are consistent with BA.1 being antigenically distinct from the ancestral and Delta strains (K. van der Straten K et al., unpub. data, https://doi.org/10.1101/2022.01.03.21268582). A boost occurred in preexisting SARS-CoV-2 neutralizing antibodies to ancestral and Delta but not in cross-reactivity to Omicron, probably because more epitopes are shared between ancestral and Delta than between those strains and Omicron. Serologic data from humans suggest that >3 exposures to ancestral strains as infection or vaccination or a combination are needed to induce cross-reactive antibodies to BA.1 (*7*). Although data from antigenic cartography using human serum suggest that BA.2 is antigenically closer to the ancestral and Delta strains (A. Rössler et al., unpub. data, https://doi.org/10.1101/2022.05.10.22274906), we did not detect cross-reactive neutralizing antibodies after primary infection with ancestral and Delta strains. Protection from replication of the Omicron BA.1 strain despite the lack of cross-reactive neutralizing antibodies may be attributable to mucosal immunity or T-cell responses in ancestral strain–infected and Delta-infected mice (*8*).

Appendix Additional information about SARS-CoV-2 Omicron BA.1 challenge after ancestral or Delta infection in mice.
