# Supplementary material for: SARS-CoV-2 Omicron BA.1 Challenge after Ancestral or Delta Infection in Mice
Source: Emerg Infect Dis. 2022 Nov;28(11):2352–5. doi: 10.3201/eid2811.220718 (PMC9622237; doi:10.3201/eid2811.220718)
Supplement: Appendix — Additional information about SARS-CoV-2 Omicron BA.1 challenge after ancestral or Delta infection in mice. [file 22-0718-Techapp-s1.pdf]

Article DOI: https://doi.org/10.3201/eid2811.220718

SARS-CoV-2 Omicron BA.1 Challenge after Ancestral or Delta Infection in Mice

Appendix

Detailed Methods

Six-to 9-week old female K18-hACE2 transgenic mice (Animal Resources Centre, Australia) were lightly anesthetized and inoculated intranasally with 10^4 50% tissue culture infectious dose (TCID50) of SARS-CoV-2/Australia/Vic/01/20 (Wuhan-1 like SARS-CoV-2 (ancestral)), SARS-CoV-2/Australia/Vic/18440/2021 (Delta) and SARS-CoV-2/Australia/NSW/RPAH-1933/2021 (Omicron BA.1) variants in 50 μL (Appendix Figure, panel A). All animal experiments were conducted in a certified Physical Containment Level 3 (PC3) facility AgriBio, Centre for AgriBioscience, Bundoora, Australia, and approved by the Animal Welfare committee at the University of Melbourne. Groups of 5 mice were monitored daily for weight loss, and mortality was recorded over a period of 14 days. Mice that lost 20% of original bodyweight were sacrificed according to the Institutional Animal Care and Use Committee guidelines. On days 2, 4 and 7 post-infection (p.i.), groups mice (n = 5) were euthanized, and the nasal turbinates (NTs), lungs and brains were harvested, homogenized and titrated in Vero (ancestral and Delta variant) or Vero-TMPRSS2 cells (Omicron BA.1). All the mice infected with ancestral or Delta variant lost weight and died by day 7 p.i., whereas mice inoculated with Omicron BA.1 did not lose weight or die (Appendix Figure, panel B). The kinetics of replication of ancestral, Delta and Omicron BA.1 variants in the NTs, lungs and brain are shown in (Appendix Figure, panel C). Ancestral and Delta strains replicated to high titers in the upper and lower respiratory tract of mice. Virus titers in NTs and lungs peaked at $10^{6.3}$ and $10^{7.5}$ $TCID_{50}$/organ for Ancestral at day 2 p.i., and at $10^{6.2}$ and $10^{6.3}$ $TCID_{50}$/organ for Delta variant, respectively. In both virus infections, the titers declined by day 7 p.i. with mean titers ranging from$10^{1.1}$ to $10^{2.6}$ $TCID_{50}$/organ in the upper and lower respiratory tract, respectively (Appendix Figure, panel C). In contrast, increasing titers of ancestral and Delta viruses from day 2 to 7 p.i. were found in the brains of K18-hACE2 transgenic mice, in accordance with previous reports

(*1*,*2*). All mice intranasally infected with either ancestral or Delta viruses had peak titers in the brain on day 7 p.i, when virus in the lower and upper respiratory tract were absent or low. Primary BA.1 infection in K18-hACE2 mice was not associated with weight loss or mortality, and virus titers achieved in the NTs and lungs were 1000 to 10,000-fold lower than following ancestral and Delta virus infections. Indeed, mean virus titers in NTs and lungs peaked at $10^{2.9}$ and $10^{3.9}$ $TCID_{50}$/organ for BA.1 at days 2 and 4 p.i., respectively, with no virus detected in the respiratory tract by day 7 p.i.. Virus replication was only observed in the brain of one mouse infected with BA.1 ($10^{1.3}$ $TCID_{50}$/organ) on day 7 p.i. (Appendix Figure, panel C).

References

1. Rathnasinghe R, Strohmeier S, Amanat F, Gillespie VL, Krammer F, García-Sastre A, et al. Comparison of transgenic and adenovirus hACE2 mouse models for SARS-CoV-2 infection. Emerg Microbes Infect. 2020;9:2433–45. PubMed https://doi.org/10.1080/22221751.2020.1838955

2. Zheng J, Wong LR, Li K, Verma AK, Ortiz ME, Wohlford-Lenane C, et al. COVID-19 treatments and pathogenesis including anosmia in K18-hACE2 mice. Nature. 2021;589:603–7. PubMed https://doi.org/10.1038/s41586-020-2943-z

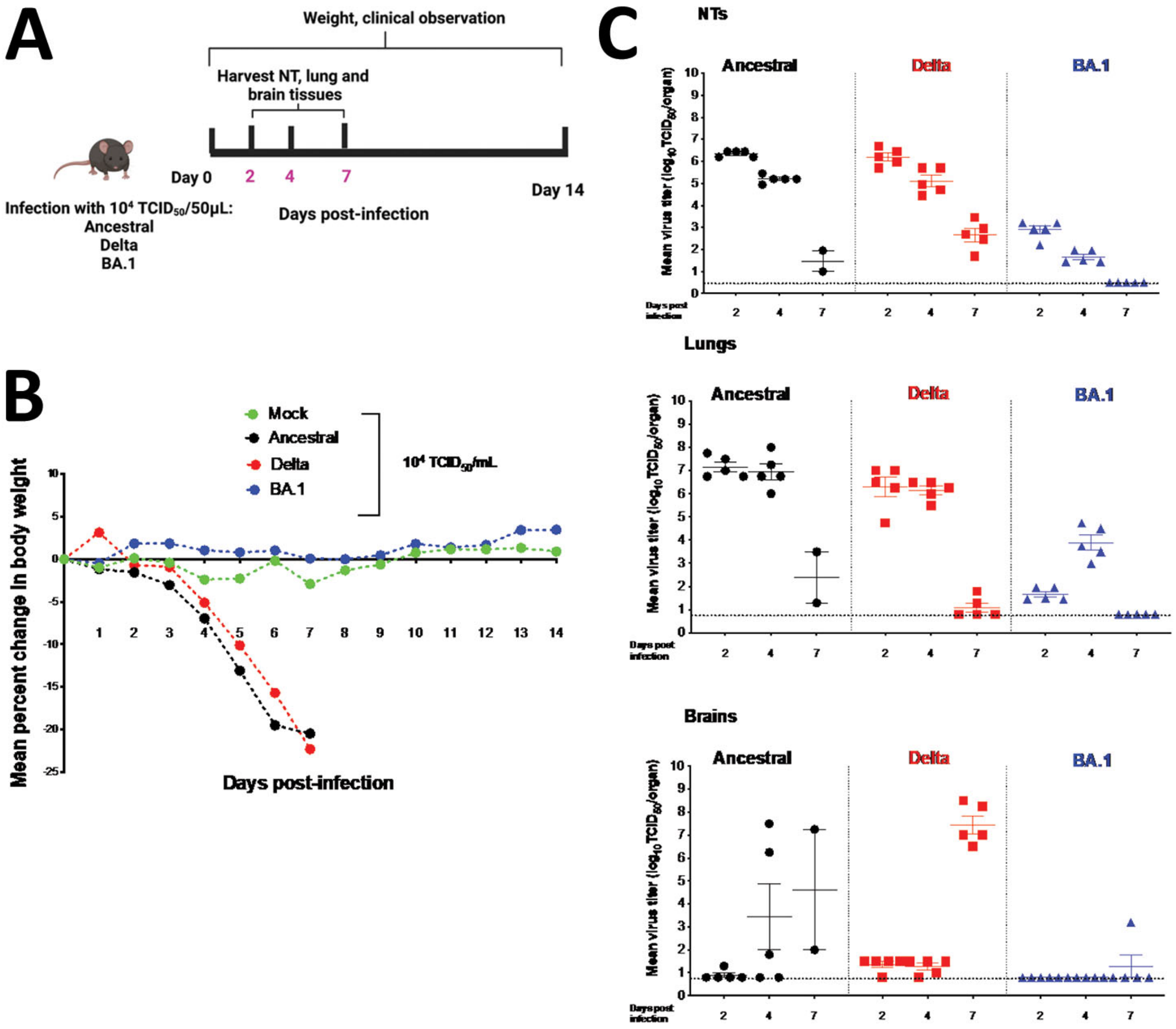

Appendix Figure. (A) Flowchart of six-to 8-week-old female human ACE2-K18 transgenic mice infected with Omicron BA.1, ancestral or Delta viruses. (B) Weight loss in mice inoculated intranasally with 50 µl containing 10^4 $TCID_{50}$ of Omicron BA.1, ancestral or Delta viruses. Animals were monitored daily for weight loss, and mortality was recorded over a period of 14 days. Mice were euthanized when they lost 20% of their original bodyweight. (C) Replication kinetics of Omicron BA.1, ancestral and Delta viruses in mice following intranasal infection of 10^4 $TCID_{50}$/virus. Virus titers in the nasal turbinates (NTs), lungs and brains of 5 mice per group sacrificed on days 2, 4, and 7 post-infection are expressed as log_{10} $TCID_{50}$/mL (NTs) and log_{10} $TCID_{50}$/organ (lungs and brains). Horizontal bars represent mean titers, and symbols represent titers from individual mice. The dashed horizontal line indicates the lower limit of detection, $10^{0.5}$ $TCID_{50}$ per mL for the NTs and $10^{0.8}$ $TCID_{50}$ per organ for lungs and brains.
